# Therapeutic Notes

**Published:** 1901-01-05

**Authors:** 


### THERAPEUTIC NOTES.

### URÆMIA.

In chronic kidney disease, where uræmia is feared, there is often a risk in giving hypodermic injections of pilocarpin, but some writers think this may be avoided if small repeated doses are taken by the mouth. It is claimed that the skin gradually becomes moist, and the general state is improved without the risk of collapse or of œdema of the lungs, and at any rate the action can be watched, and any excessive effect neutralised if necessary by atropine. The following formula may be employed:

℞ Pilocarpini gr. i.

Acidi hydrochlor. dil ʒi.

Aquæ destillat. ℥ii.

M. S.—A teaspoonful every three or four hours.—*Med. Record*, August 4th.

### INSOMNIA.

Professor Königshöfer, of Stuttgart, has used dormiol with great success before and after operations and in hysterical patients, and found it a safe and powerful hypnotic in doses of 6 to 12 minims of a 10 per cent. solution. Meltzer confirms his statements as to the absence of any unpleasant after-effects, even when given in dozes of 24 to 36 minims. It appears to be useless in extreme excitement or severe pain. Otherwise, sleep generally comes on in half an hour to an hour, and lasts five to eight hours. Schultze has given over 1,000 doses, which generally amounted to 18 minims each, with marked success, and with no disturbance of the digestion or general health. It apparently closely resembles chloral hydrate in its action, but is without its drawbacks.—*Mercks Archives*, August.

